# Surgical Diseases of the Kidney and Ureter

**Published:** 1902-03

**Authors:** 


**Surgical Diseases of the Kidney and Ureter.** By Henry Morris, M.B. Two Volumes. Vol. I. Pp. xii., 682. Vol. II. Pp. vii., 670. London: Cassell and Company, Limited. 1901.

We cannot speak too highly of this work, every page of which is impressed with evidences of exhaustive research and well-balanced judgment. Although Mr. Morris's opinion may be regarded as authoritative in renal surgery, a special charm of the monograph is the liberal manner in which the work of others has been recognised and dealt with. The surgeon may thus refer to any section and find the best of everything recorded with commendable consistency and completeness. The labour expended in the production must have been immense, and we congratulate the author upon a work which will be the standard one for many years to come. It would be scarcely possible, if not invidious, to offer any criticism of so masterly a work. The reliability of the X-rays in the detection of calculi has doubtless increased since the text was written, but Mr. Morris rightly calls attention to the fallacies of this means of diagnosis in certain cases; and we think he takes a somewhat conservative view of the value of cystoscopy and ureteral catheterization—though we agree with him that it would be a misfortune if these measures were too widely adopted, on account of the difficulties and risks. Under nephropexy we have not noticed reference to the method by suspension with gauze. Mr. Morris's book will prove a necessity to everyone interested in the surgery of the kidney and ureter, and is an ornament of great value to British literature.

